# Saline and Arid Soils: Impact on Bacteria, Plants, and Their Interaction

**DOI:** 10.3390/biology9060116

**Published:** 2020-06-02

**Authors:** Elisa Gamalero, Elisa Bona, Valeria Todeschini, Guido Lingua

**Affiliations:** 1 Dipartimento di Scienze e Innovazione Tecnologica, Università del Piemonte Orientale, Viale T. Michel 11, 15121 Alessandria, Italy; elisa.gamalero@uniupo.it (E.G.); guido.lingua@uniupo.it (G.L.); 2 Dipartimento di Scienze e Innovazione Tecnologica, Università del Piemonte Orientale, Piazza San Eusebio 5, 13100 Vercelli, Italy; valeria.todeschini@uniupo.it

**Keywords:** salinity, aridity, microbial community, rhizosphere, PGPB, plant stress

## Abstract

Salinity and drought are the most important abiotic stresses hampering crop growth and yield. It has been estimated that arid areas cover between 41% and 45% of the total Earth area worldwide. At the same time, the world’s population is going to soon reach 9 billion and the survival of this huge amount of people is dependent on agricultural products. Plants growing in saline/arid soil shows low germination rate, short roots, reduced shoot biomass, and serious impairment of photosynthetic efficiency, thus leading to a substantial loss of crop productivity, resulting in significant economic damage. However, plants should not be considered as single entities, but as a superorganism, or a holobiont, resulting from the intimate interactions occurring between the plant and the associated microbiota. Consequently, it is very complex to define how the plant responds to stress on the basis of the interaction with its associated plant growth-promoting bacteria (PGPB). This review provides an overview of the physiological mechanisms involved in plant survival in arid and saline soils and aims at describing the interactions occurring between plants and its bacteriome in such perturbed environments. The potential of PGPB in supporting plant survival and fitness in these environmental conditions has been discussed.

## 1. Arid and Saline Soil

Drought and salinity are the most important abiotic stresses hampering crop growth and yield. Many environmental and anthropogenic factors influence the soil salinization and its aridity: the scarce vegetation cover associated with low evapotranspiration, drainage, irrigation water quality, agronomic practices (e.g., fertilization and pesticide application), saline groundwater intrusion, marine floods, and shallow aquifers that facilitate salt accumulation. In general, soil salinity is classified as “primary”, if salt is accumulated by natural process and “secondary” if the accumulation is a consequence of the management of natural resources [1,2]. Prolonged exposure to meteorological agents such as precipitations and winds can result in semi-arid and arid areas with naturally saline soils [3]. In these areas, rainfall is not enough to leach soluble salts from the soil [4]. This kind of salinity, called “transient salinity”, falls into one of the three types of the salinity examined by Rengasamy [2], together with “salinity associated to groundwater” (also known as “dryland salinity”) and “irrigation salinity”.

Reclamation of soil salinity and sodicity includes a combination of different physical, chemical (amendments as CaCl_2_·2H_2_O and CaSO_4_·2H_2_O), hydrological, and biological (biofertilizers and genetic engineering) methods aimed to remove Na^+^ from colloids and from the root zone, in order to allow plant cultivation in arid and semiarid areas [5].

Soil salinity is a global problem that severely affects more than 800 million hectares of arable land worldwide [6,7]. A soil is defined “saline” if the electrical conductivity of its saturation extract (ECe), measured at 25 °C, is over 4 dS m^−1^ [8], its salt concentration is ≥0.25 % and pH < 8.5; it must be considered that salinity of sea water ranges between 3 % and 4 % [9]. Salinity in soil is mainly due to NaCl, but also carbonates and sulfates of Ca, Mg, and K can be accumulated in large amounts. When soil clay particles absorb sodium until leading to ECe lower than 4 dS m^−1^ and pH > 8.5, many physico-chemical problems begin to occur, and the soil becomes “sodic” [9].

An excess of soluble salt accumulation in arable soils negatively affects agriculture, with a heavy economic impact due to low crop productivity, with repercussions on human nutrition and health [9]. Arid areas are considered the largest biome on Earth and cover 41% of the total Earth area in the world [10]. More recent estimates increase this percentage to 45% [11], highlighting the development of new vast and increasingly arid territories located in Alaska, Siberia, and towards lower latitudes, such as in central-Southern Asia, where only China represents the 55% of the lands newly exposed to aridity in Asia, or 31% worldwide. Southeast Africa and the North, including Morocco, Algeria, and Libya, have also seen an increase in drought. In North and Central America, the largest expansions of arid areas occur in the United States, Canada, and Mexico, while in South Argentina, shows an increase in dry areas equal to about 750,000 km^2^, or 27% of the national area, while in Chile, they exceed 50,000 km^2^. Australia has also been affected by an expansion of total arid lands of over 280,000 km^2^ (4% of the total area). In a study conducted in 2017, it emerged that the part of the territory with high or very high risk of desertification had increased, in less than a decade, by 177,000 km^2^ [12].

Studies carried out between 1999 and 2013 show that around 34% of European ecosystems have undergone significant alterations in their functioning. In Europe, salinization affects about 1 million hectares of lands, especially those of Mediterranean countries [1]. These include vast regions in Ireland, Germany, Poland, Greece, Italy, Spain, and Turkey. On average, 2.4% of European ecosystems have undergone a strong negative variation every year, corresponding to dry years and very dry seasons; 2005 was the year with the highest rate of sudden changes [13].

This work is an overview of the main processes occurring in the microbial community present in arid and saline perturbed soils. In particular, we focused our attention on arid environments, on the PGPB role, and on the bacterial communities occurring in saline soil. Moreover, the interaction between rhizobacteria and plants grown in arid soil and the plant responses to salt were explored.

## 2. Rhizosphere: A Scenario for Plant-Microbe Interaction

It was 1904 when Lorenz Hiltner [14] proposed the term “rhizosphere” for the first time, indicating the portion of soil attached to the root, in which the microbial density was higher than in uncultivated soil. Several definitions of rhizosphere were proposed afterwards, but the Hiltner one remained universally accepted [15].

The higher bacterial density observed by Hiltner in the rhizosphere is due to the release by the root of different compounds in solid, liquid, or gaseous forms through the secretion process known as root exudation or rhizodeposition. The flow of photosynthetic carbon that spreads from the root to the soil consists in the secretion activity of exudates such as sugars, organic acids, vitamins, mucilage, lysates, and many other substances [16]. There is a general consensus on the fact that about 11% of the total fixed C (or about 27% of the C allocated to roots) is released in the surrounding soil. However, these values, as well as the composition of the root exudates, vary according to the plant species, age, or plant nutritional status and to the exposition of environmental (biotic and abiotic) factors [16,17,18]. A direct link subsists between the composition of the root exudates and the composition of the associated microbiota. In fact, such molecules can play an important role as chemoattractants and repellents for bacteria, fungi, and viruses, which in turn, can affect plant growth, yield, health, and the nutritional value of seeds and fruits [19,20].

This concept is known as the rhizosphere feed-back loop and it is a dynamic process. The rhizodeposition modifies the environment surrounding the root, and the soil microbiota sense these variations. Then, different bacterial populations respond to the environmental modification, induced by the release of root exudates, modifying their own physiology. This leads to a selection favoring the bacterial populations best adapted to this new condition. This is at the base of the coevolution between the plant and its microbiota [21]. As a result, the induced shift in the microbial community composition and activity leads to different effects on plant development. Microorganisms can stimulate rhizodeposition and establish relationships for nutrient acquisition [21]. They can produce and consume trace gas, influence acidity, water availability, and hydrophobicity of soil. Moreover, they can degrade xenobiotic compounds and chelate metals [22]. On the other hand, there are also harmful parasitic interactions for plants and even fatal ones. Soil borne diseases in agricultural land reduce both the yield of the crop product and its quality [23], thus leading to lower financial returns. As an example, yield losses due to pathogenic organisms in rice ranged from 24% to 41% in Asia [24]; similarly, an amount ranging from 5% to 96% of potato production in France is lost due to plant diseases [25].

The plant selects the most suitable species-specific microbial composition, but the community structure and stability also depend on the type of soil [26]. In fact, different plant species host distinct microbial communities and specific microbial taxa are preferentially associated with specific plant species [26]. It is also true, however, that many soil bacterial taxa can be cosmopolitan and able to associate with a wide range of plant taxa [22].

As easily understood, the relation between plant development and its associated microbiota is so intimate that we referred to the plant as a superorganism or a holobiont, resulting directly from very complex plant-microorganisms and microbe-microbe interactions [27,28].

## 3. Plant Growth-Promoting Bacteria (PGPB)

As previously stated, following the release of root exudates, a large amount of microorganisms colonize plant roots with a different degree of intimacy (ranging from bacteria living in the rhizoplane to endophytic bacteria, who enter the root and occupy the internal plant tissues or even cells) and with different effect of plant growth and health. Among them, plant growth-promoting bacteria (PGPB) are typically defined as symbiotic or free-living bacteria in soil, able to efficiently colonize the root and induce a beneficial impact on the host plant. The major taxonomic groups of PGPB belong to Proteobacteria and Firmicutes [29,30]. *Bacillus* sp. is the most known genus of Firmicutes inducing plant growth promotion. Strains ascribed to the genera *Rhizobium*, *Azospirillum*, *Burkholderia*, *Achromobacter*, *Pseudomonas*, *Acinetobacter*, *Serratia*, *Pantoea*, *Enterobacter*, and *Rahnella* are the most representative of the phylum Proteobacteria reported to behave as PGPB [31].

**Figure 1:**
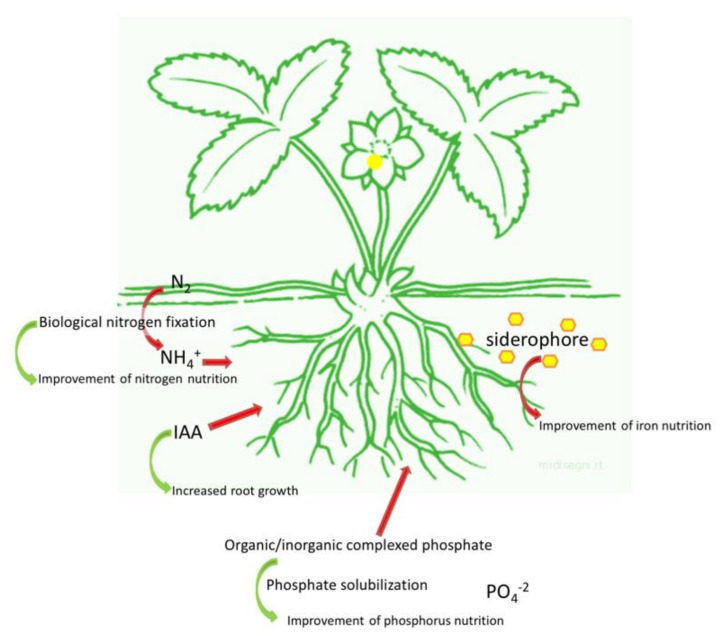
Overview of the main direct mechanisms used by PGPB. Improvement of mineral nutrition via nitrogen fixation, phosphate solubilization, and iron chelation, as well as the modulation of phytohormones levels.

The stimulation of plant growth occurs by direct and indirect mechanisms. While direct mechanisms include the facilitation of nutrient acquisition and the modulation of plant hormone levels, indirect mechanisms are based on the suppression of phytopathogenic microorganisms responsible for soil borne diseases, thereby acting as a biocontrol agent [32]. In agreement with the topic of the review, a brief overview of the direct mechanisms (Figure 1) is reported in the next paragraph.

### 3.1. Stimulation of Plant Growth Induced by Direct Mechanisms

PGPB-dependent plant growth promotion can occur through the improvement of plant mineral nutrition, especially as far as nitrogen (N), phosphate (P), and iron (Fe) uptake are concerned [33]. This enhancement of P, N, and potassium (K) uptake in plants, especially in the shoot, can reduce the accumulation of Na^+^ and Ca^+^ and this is an important process for the plants growing in saline/arid soil.

Nitrogen is one of the most important nutrients for plant growth and yield: this element is very abundant in the atmosphere as N_2_, but unavailable to plants in such form. However, N_2_ can be converted in ammonia with the help of specific groups of microorganisms able to carry out the biological N fixation (BNF) through the activity of the nitrogenase [34,35]. N-fixing microorganisms can be free living or symbiotic. Free-living microorganisms include genera as *Azospirillum* and *Azotobacter* [36]. Among the symbiotic bacteria, the members of the family Rhizobiaceae (*Rhizobium*, *Bradyrhizobium*, *Sinorhizobium*, *Azorhizobium*, and *Mesorhizobium*) can establish symbiotic relationships with legume, while bacteria belonging to *Frankia* genus can be associated with other plant families among which, Betulaceae (*Alnus glutinosa* for example) [37].

The close dialogue between rhizobia and the leguminous plants, mediated by the bacterial lipochitooligosaccharides (Nod factors) and plant flavonoids, leads to the formation of root nodules [36,38]. The different zones of the nodule are readily colonized by rhizobia cells that differentiate into bacteroids with diverse size and metabolic activity [38]. Inside the nodule, N fixation is driven by the nitrogenase enzyme that is composed by two subunits: the dinitrogenase reductase, with iron as cofactor, and dinitrogenase, having iron and molybdenum as cofactors [35]. Nitrogenase is oxygen sensitive, but rhizobia are strictly aerobic microorganisms: the trade-off solution is offered by the synthesis of leg-hemoglobin, a protein binding oxygen with high affinity, therefore ensuring nitrogenase activity, as well as bacterial viability [39]. Moreover, the nodule external structure, made by compact layers of cells, is characterized by a low permeability to O_2_ and is involved in reducing oxygen availability [40].

Nitrogen accumulation in crop plants can be reduced by salinity since an increase in the uptake of Cl^−^ occurs together with the decrease in shoot NO^−3^ of many crops. Moreover, soil salinity may hamper symbiotic N_2_-fixation by reducing the density of rhizobia in the soil and their efficiency in colonizing root hairs. Even though the symbiosis is established, the stunted growth of the host plant in such condition leads to a reduction of the amount of photosynthate transferred to the nodule [41].

Non-symbiotic diazotrophic bacteria are able to fix N in non-leguminous plants: *Azospirillum* and *Azotobacter* are the most studied genera among them. *Azospirillum* is a Gram-negative, non-nodule-forming aerobic N-fixing bacterium belonging to the family Spirillaceae [42], while *Azotobacter* is free-living, aerobic, photoautotrophic non symbiotic bacterium assigned to the family Azotobacteriaceae. The most beneficial species are *Azospirillum lipoferum*/*brasilense* and *Azotobacter chroococcum/vinelandii*, respectively [43].

The mechanisms at the base of the positive effects on plant growth induced by strains of these two genera are not limited to N fixation. *Azospirillum* strains synthesize phytohormones (IAA, gibberellins, and cytokinin) and modulate root development and uptake of plant nutrients (N, P, and K). Similarly, *Azotobacter* releases vitamin B complex, different phytohormones such as gibberellins, naphthalene acetic acid, and other substances involved in biocontrol of root pathogens and promoting root growth and mineral uptake [44].

Similar to N, P is very abundant in soil, but its bioavailability is very low. P in soil is present in two main insoluble forms: mineral forms such as apatite, hydroxyapatite, and oxyapatite, and organic forms including inositol phosphate (soil phytate), phosphomonoesters, phosphodiesters, and phosphotriesters [45,46]. It has been estimated that the average P content of soil is about 0.05% (*w*/*w*): only 0.1% of this amount is available for plants [47]. As a consequence, P-based fertilizers are usually applied; however, right after the application, a fraction corresponding to 75–90% of the added P precipitates with metal-cation complexes, rapidly becomes fixed in soil [48,49,50].

In this context, phosphate solubilizing bacteria represent a sustainable and environment friendly solution to enhance P availability for plants especially in P poor soils. Inorganic P is solubilized by the synthesis of low molecular weight organic acids such as gluconic and citric acid or by the release of protons, and the production of chelating substances. On the other hand, the mineralization of organic phosphorus occurs through the synthesis of phosphomonoesterase, phosphodiesterase, and phosphotriesterase which catalyze the hydrolysis of phosphoric esters [51,52,53,54]. Phosphate solubilization tends to be more efficient in microorganisms isolated from extreme soils (such as saline/alkaline soils and soil with nutrient deficiency) than in those coming from soils characterized by moderate conditions [47].

Iron is the fourth most abundant element on earth [55], but in aerobic soils, Fe forms insoluble hydroxides and oxyhydroxides [56], which limit its bioavailability particularly in calcareous soils [57], that cover about 30% of cultivated soils in the world. For this reason, plant Fe nutrition in these soils is one of the most important agricultural problems worldwide [57,58]. Iron is crucial for all forms of life [59]: while microorganisms grow at an optimal Fe concentration of about 10^−5^ to 10^−7^ mol·L^−1^ [60], for plants, the optimal amount of Fe ranges between 10^−9^ to 10^−4^ mol·L^−1^ [61,62].

Usually, the low bioavailability of iron is faced with application of synthetic iron chelates, such as ethylenediaminetetraacetic acid (EDTA) or ethylenediamine-*N*, *N*’-bis(2-hydroxyphenylacetic) acid (EDDHA). This kind of approach is cheap and effective but, besides their high affinity for Fe, these chelators are not biodegradable and their accumulation in the environment is of great concern [63]. The availability of Fe in saline sodic soils is usually very low [64,65] and plant growth reduction is often reported to occur in such environmental condition. The high level of Fe starvation in saline soil leads to an altered chlorophyll content of young leaves and subsequently to the appearance of Fe chlorosis symptoms. Moreover, salt stress severely affects Fe transport to shoots, since its content is usually lower in aerial organs than in roots [65].

**Figure 2:**
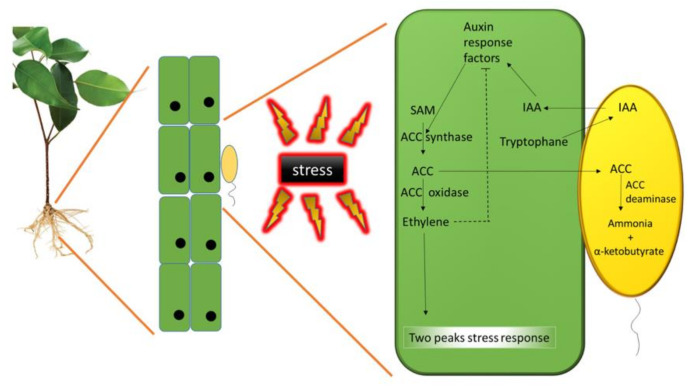
Bacterial Indole- 3-Acetic Acid (IAA) and 1-Amino-Cyclopropane-1-Carboxylic Acid (ACC) deaminase interplay: Plant ethylene is synthesized from S-adenosyl-L-methionine (SAM) and ACC through the enzymes SAM synthetase and ACC synthase, respectively. Then, ACC is converted to ethylene by ACC oxidase.

The Fe requirement is more accentuated in the rhizosphere, where the competition for this element among plants, bacteria, and fungi is higher than in bulk soil [61,66]. In order to cope with the low amount of available Fe, bacteria have evolved the synthesis of low weight molecules (between 500 and 1500 Da) called siderophores, having a high affinity for Fe^3+^. When the bacterial cell is in starvation for Fe, the synthesis of siderophores is induced and the chelators are released outside the cell. The siderophore binds Fe^3+^ and the Fe-siderophore complex, called ferrisiderophore, is recognized by a receptor localized on the cell membrane. The ferrisiderophore is taken up to a membrane receptor, allowing the internalization of this element that is subsequently transformed at the membrane level as Fe^2+^ and released inside the cell [67] (Figure 2).

Microorganisms able to synthesize siderophores are present in a large amount in the rhizosphere [68]. As a consequence, the siderophore concentration is maximum in the rhizosphere, where it reaches 0.1 μmol·L^−1^ and 1 mmol·L^−1^ for bacterial and plant siderophore, respectively [69], while a very low level is found in bulk soil (10 nmol·L^−1^) [70]. Among rhizobacteria, the use of homologous siderophores, produced by members of the same genus, is very common, while the use of heterologous ones, synthesized by bacteria belonging to different genera, is also possible [71].

Plants assimilate Fe from bacterial siderophores by different mechanisms such as chelating and releasing Fe, direct uptake of ferrisiderophores, or by a ligand exchange reaction [72,73]. In plants, active Fe uptake occurs mainly through two strategies [74]. The first strategy (Strategy I) occurs typically in dicotyledonous and nongraminaceous monocotyledonous plants, and it is based on the acidification of the rhizosphere by H^+^ excretion, leading to the reduction of Fe^3+^ to Fe^2+^ by a plasma membrane-bound reductase and its transport inside root cells [75,76,77]. The second strategy (Strategy II), is mainly used by grasses and graminaceous plants, and it is based on the synthesis of Fe chelators (phytosiderophores) and on the uptake of the Fe–phytosiderophore complex in root cells mediated by specific transporter molecules [78,79]. Therefore, siderophores are involved both in suppressing the growth of phytopathogenic microorganisms via competition for the available Fe and in stimulation of plant growth by supplying the plant this nutrient.

Rhizobacteria can produce phytohormones and can modulate their endogenous amount in plants. It is well known that phytohormones play a crucial role in plant growth and development [80,81,82].

It has been estimated that about 80% of microorganisms living in the rhizosphere of various crops are able to synthesize and release auxins (IAA) by five biochemical pathways, depending or not on tryptophan as precursor [83,84]. IAA regulates both plant growth and defense responses: although its main role is the modulation of cell division, extension, and differentiation. IAA is also involved in seed germination, enhancement of root branching or elongation, regulation of responses to light, gravity, fluorescence, photosynthetic pigment biosynthesis, metabolite production, and resistance to environmental stresses [82,83,85]. In fact, when plants are exposed to stressful conditions, a regulation of plant phytohormones finalized to the adaptation to the stressor is often observed [86].

Plant reactions to exogenous IAA vary according to the endogenous concentration of this phytohormone, the plant species, its phenological stage, sensitivity, and the tissue involved [87]. For example, root elongation in *Arabidopsis thaliana* seedlings was stimulated only by exogenous concentrations ranging from 10^−10^ and 10^−12^ M [88]. Moreover, it should be considered that plants respond to a total pool of IAA that is composed by the endogenous fraction as well as by the IAA synthesized by soil microorganisms. The impact of bacterial IAA on plant development ranges from positive to negative effects according to the amount of IAA available to the plant and to the sensitivity of the host plant to the phytohormone. Therefore, the amount of IAA synthesized by the plant itself determines if the bacterial IAA will stimulate (optimal level) or suppress (suboptimal level) plant growth [84]. Therefore, the amount of IAA driving the growth of the plant has to be carefully regulated. In order to avoid inhibitory effects from over dosage, plants possess neutralization mechanisms to control IAA excess, conjugating IAA with sugars, amino acids, or peptides [89].

### 3.2. Increasing Plant Tolerance to Stress: 1-Amino-Cyclopropane-1-Carboxylic Acid (ACC) Deaminase and Exopolysaccharide Synthesis

Being sessile organisms, plants are characterized by a high level of physiological plasticity, which ensures a wide range of protective responses and enables them to survive a variety of different environmental stresses that is now called the “plant like-immune system” [90,91]. Ethylene is a gaseous plant hormone driving several aspects of plant growth, such as fruit ripening, flower senescence, leaf and petal abscission [92,93], and plays a key role in plant defense responses to stress and to symbiotic relationship [94,95,96].

In fact, the term “stress ethylene” [97] is usually used to indicate the rise in the ethylene level after plant exposure to environmental stresses such as: extremes of temperature, high light, flooding, drought, heavy metal, organic pollution, radiation, wounding, insect predation, high salt, and various pathogens including viruses, bacteria, and fungi [98].

Ethylene biosynthesis occurs in all higher plants via a methionine dependent pathway, in which methionine is converted to S-adenosyl-L-methionine (SAM) by the SAM synthetase. SAM become the substrate for ACC synthase that converts it in ACC, which is the immediate precursor of ethylene. Then, ACC oxidase catalyzes the conversion of ACC to ethylene, carbon dioxide, and cyanide (Figure 2) [99].

The amino acid tryptophan is exuded by plant cells, taken up by PGPB living around the roots, and used to synthesize IAA. This bacterial IAA is taken up by the plant cells and, together with the plant’s endogenous pool of IAA, stimulates an auxin signal transduction pathway which results in cell growth and proliferation, and in increased transcription of the gene for ACC synthase, eventually yielding an increased concentration of ACC. As a consequence, ACC is synthesized on SAM as substrate favoring the production of ethylene and leading to a two peaks plant stress response. The newly produced ACC is partly exuded from plant roots, taken up by the bacteria, and converted by ACC deaminase to ammonia and α-ketobutyrate. As a result, the amount of ethylene produced by the plant is reduced. Alleviation or exacerbation of stress depends on the plant species, its age, and the nature of the stress itself [100]. The answer of the plant to environmental stress involving ethylene has been explained by Glick et al. [101]. In this model, a few hours after the onset of a stress, an initial small peak of ethylene rises. This peak is considered to be beneficial since it triggers a protective response by the plant, such as the transcription of pathogenesis-related genes and induction of acquired resistance [100,102]. When the stress become chronic or more intense, the transcription of the ACC synthase genes occurs some days later, and another ACC is synthesized leading to a second, much larger ethylene peak. This second peak induces processes such as senescence, chlorosis, and abscission; therefore, the growth and the survival of the plant are hampered. A fraction of the ACC synthesized by this two-step process is released from seeds or plant roots, taken up by the bacteria, and converted to ammonia and α-ketobutyrate by the enzyme ACC deaminase [103]. As a consequence, the amount of ethylene produced by the plant after the stress is reduced and does not reach inhibitory levels. Various environmental stresses can also either increase the synthesis of IAA or stimulate the transcription of the gene for ACC synthase. As the amount of ethylene in a plant increases, the transcription of auxin response factors is inhibited. In the absence of bacterial ACC deaminase, by limiting transcription of auxin response factors, ethylene hampers both cell growth and IAA stimulation of the synthesis of additional ethylene. In the presence of bacterial ACC deaminase, less ethylene is formed because some of the ACC is degraded by ACC deaminase. When ACC deaminase is synthesized, transcription of auxin response factors is not suppressed, and IAA can stimulate cell growth and proliferation without inducing a rise of the ethylene level. In this model, ACC deaminase both decreases ethylene inhibition of plant growth and allows IAA to boost plant growth, both in the presence and absence of plant stress (Figure 2). Therefore, plants hosting bacteria able to synthesize ACC deaminase and exposed to stressful conditions result to be more tolerant to stress and their growth is comparable to that of plants grown in normal conditions. In the same way, bacteria synthesizing this enzyme favor the establishment of symbiosis with rhizobia (with an increased nodulation) [104,105,106] or mycorrhizal fungi [107,108,109], thus indirectly improving both plant growth and health.

The term exopolysaccharide (EPS) was coined in 1972 by Sutherland [110] in order to indicate the different types of layers occurring outside the bacterial cell wall and represents the 40–95% of the bacterial mass. Bacterial EPSs fall in two categories: slime EPSs and capsular EPSs both composed by a mixture of polysaccharides, proteins, nucleic acids, and lipids [110] whose weight reach 10–30 KDa [111]. The water content in the EPS reaches ∼97% and is considered as a sink of water involved in protection of bacteria from desiccation [112].

Due to their chemical and physical properties, EPSs found application in biotechnology industries as gelling, stabilizing, thickening, coagulating, film forming agents for detergents, textile, paper, paints, adhesive, beverages, and mainly food production [113,114]. Because of their impact on human health, bacterial exopolysaccharides, and especially those produced by lactic acid bacteria, are considered as beneficial since their modulation of the immune system, cholesterol-glucose lowering ability, antitumoral activities, and prebiotic effect [115]. Many PGPB belonging to the *Pseudomonas*, *Azospirillum*, and *Rhizobium* genera are reported to form strict association with plant roots through the formation of an EPS layer that is involved in biofilm building [116]. Moreover, during drought or salinity stress, EPS synthesis in bacteria is higher than in non-stressful conditions [117]. Inoculation of plants with EPS synthesizing PGPB increase plant resistance to drought and excess of salt in soil [118]. While both these stresses lead to severe change of the soil chemical and physical properties, making it unsuitable for an optimal crop productivity, the bacterial EPS produced in soil and all around the roots is crucial in maintenance of micro and macro aggregates and contributes to the formation of a site where the soil microbial process occurs, efficiently leading to an optimal flow of nutrient, water, and ions to the plant root systems, also in conditions of low water availability [119].

## 4. Effects of Salinity on Plants and Plant Responses

On the basis of their ability to grow in a saline environment, plants are classified into *glycophytes* and *halophytes* [120,121,122], the former are unable to grow in saline soils, the latter are salt tolerant. About 98% of the plants are glycophytes: they tend to exclude sodium, maintaining low levels of Na^+^ in their tissues (0.2–2.0 g Kg^−1^). Therefore, these plants are very salt-sensitive and are heavily affected by the presence of salt in the soil [122]. Some representative genera are *Citrus* and *Persea*, besides different species of the family Fabaceae (e.g., bean, chickpea and pea) and Poaceae (e.g., maize and rice). Only a very small number of glycophytes, among which *Medicago sativa*, *A. thaliana*, *Gossypium* spp., *Solanum lycopersicum*, and *Hordeum vulgare* are able to develop a certain degree of tolerance in response to the changing of soil salinity, showing reduced sensitivity to salt [123]. Halophytes represent only 2% of the total flora and generally tolerate high salt concentrations, therefore they are considered salt-resistant plants [121]. Halophytes evolved a series of adaptative traits that allow them to complete their life cycle under elevated salinity (more than 200 mM NaCl) [124]. Three main categories of halophytes are known: obligate, facultative, and preferential halophytes. Obligate halophytes require salt concentration in a range between 200–300 mM NaCl to reach optimal growth and can tolerate seawater salinity. The genus *Salicornia*, *Suaeda*, *Halimione*, *Atriplex*, *Tamarix*, and *Petrosimonia* are included in this group. Facultative halophytes do not necessarily require salt for their growth, therefore they can live also in soil devoid of salt [125]. *Chenopodium quinoa* has been classified as facultative halophyte, able to grow in marginal soils such as saline and arid ones; its ability to tolerate these extreme conditions could be improved by the use of halotolerant rhizobacteria as bioprotectors [126]. Preferential halophytes can grow at a relatively higher concentration of salt in soil, in respect to glycophytes. On the basis of anatomical adaptations in response to salinity, halophytes are also divided into extreme-halophytes (mainly succulent plants), able to store water in plant aerial tissues or organs and meso-halophytes, that include species with expanded endodermis, aerenchyma and bulliform cells, with intermediary anatomical adaptations between extreme-halophytes and glycophytes [127].

Similar to other important abiotic stresses, among which aridity, salinity induces a variety of responses in plants including morphological, physiological, biochemical, and molecular changes. It is well known that water deficit and salt stresses are strictly associated, since the negative effects of salt stress on plants have been mostly associated to the osmotic imbalance due to the decrease of water potential in soil, following by decreased water absorption by the plant root system. Therefore aridity and salinity can induce similar negative effects on plant growth and physiology [6,128,129,130,131], in particular, damaging membrane components (lipids and proteins), inducing seed dormancy, reducing germination rate, root elongation, shoot biomass, leaf expansion, stomatal conductance, and photosynthetic efficiency, leading to a substantial loss of crop productivity and yield, resulting in significant economic damage [3,9,130,132,133]. It has been demonstrated that plant fitness and productivity are closely related to the seed germination rate, a crucial phase of plant growth, strongly affected by many adverse environmental factors such as soil salinity. Cuartero and Fernandez-Muñoz [134] showed that tomato seed germination was strongly inhibited (from 50% to 100%) at 80 and 190 mM NaCl, respectively, if compared to that of seeds grown in not disturbed soil. Similar results were reported, at different salt concentrations, in many other plant families such as Poaceae [135,136], Fabaceae [137], and Brassicaceae [138,139]. Besides the seeds, other plant organs can be severely affected by salt toxicity. Roots are embedded in the soil and they are the first organ to face salinity [129]. For this reason, growth reduction is greater at root level than at shoot. An increased root to shoot ratio was reported under salt stress [7]. However, it was observed that the extent of salt-induced damage could depend also on plant/organ developmental stage [140]. In fact, it has been shown in some studies that high concentrations of salt can induce the expression of genes which, in addition to conferring a different ability of the plant to respond to stress, are also involved in regulating the development of the plant in different phenological stages such as flowering, seed germination, root growth /branching, and leaf morphology [7,141,142]. As suggested by Munns [143], mature leaves are more affected by salinity than young leaves: they present necrotic tissue (sign of premature senescence), probably due to the imbalance of the detoxification process in vacuole. Several anatomical changes (such as variation in palisade and spongy parenchyma thickness; increased intercellular spaces; damage of chloroplast ultrastructure and increased number of plastoglobules) were recorded in leaves of plants exposed to salt stress [7].

At the cellular level, the primary site of salt injury is the plasma membrane [144,145]. The so called “salt shock” induces changes in the membrane lipids and proteins due to the fact that salts are found in the solution in ionic form. The positive charges of the ions can break the electrostatic bonds present between the polypeptidic chains, increasing hydrophobic interactions and leading to loss of protein/membrane functionality. The alteration of the ionic flow across membrane, has a negative impact on important processes such as photosynthesis, respiration, and protein metabolism. High concentrations of Na^+^ and Cl^−^ can also influence the membrane potential and the activities of membrane bound enzymes, besides the uptake of other minerals, therefore resulting in a nutritional imbalance for the plant. Different studies showed that salinity decreases the availability of nutrients and their assimilation in plants [3,146]: for example, Na^+^ ions can compete with other cations (such as K^+^) for membrane transporters, causing toxic accumulation of Na^+^ in the cell [147,148], decreasing the uptake of K^+^. The effects of salt stress can also be reflected in variations of membrane permeability, which has been reported as an effective selection criterion for salt tolerance in different crops [145,149].

Salt-induced osmotic stress is responsible for oxidative damage caused by reactive oxygen species (ROS) as superoxide anion (O_2_^−^), hydrogen peroxide (H_2_O_2_), and hydroxyl radical (HO^•^) [3,129]. The production sites of ROS in cell are various: they are produced in mitochondria during respiration, in peroxisomes during photorespiration, and in chloroplasts during photosynthesis. Therefore, the control of ROS intracellular levels is strictly necessary to avoid serious damage to all these cellular processes [3,150]. Moreover, ROS are interconnected with other molecules, including phytohormones (e.g., abscisic and gibberellic acids), and this connection is very important in modulating plant stress responses [150,151].

The plant photosynthetic apparatus is negatively affected by ROS, especially photosystem II (PSII) and the electron transport chain [152]: salt stress induces stomatal closure (due to a decreased uptake of CO_2_) and oxidative stress, resulting in a low photosynthetic rate and in an imbalance of photosynthetic pigment biosynthesis [3,153,154].

**Figure 3:**
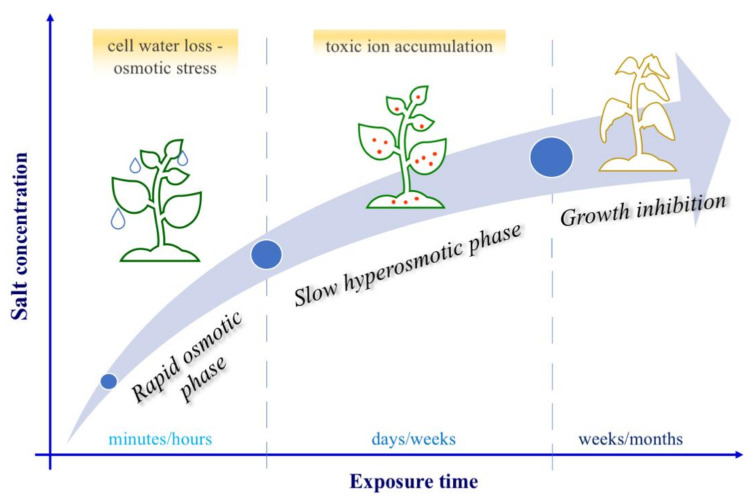
Plant responses to salt stress. Plants cope with the increase in salt concentration in different ways as the time of exposure to stress increases. Three phases are identified: a faster first reaction (minutes/hours) called rapid osmotic phase; one in the order of days/weeks called slow hyperosmotic phase; and a long term exposure (weeks/months) which leads to inhibition of growth and cell death.

The above mentioned effects of salt stress on plants largely depend on the concentration of salt in soil, on the exposure time to stress (that can vary between minutes/days to days/weeks/months) [6,155] and on the plant’s ability to absorb salt before it becomes highly toxic, strongly affecting metabolic function and plant life itself [6]. An increase of salinity in soil leads cells, in a few minutes, to lose water due to osmotic stress: this phenomenon, known as “rapid osmotic phase” is followed by a “slower (days/weeks) ion toxicity phase” (or “hyperosmotic stress phase”) (Figure 3), that is characterized by a high accumulation of toxic ions, decreasing both cell division and expansion rate [6,156]. Therefore, over time (weeks/months), the vegetative development of the different plant organs is strongly altered.

Another factor to consider is plant tolerance/sensitiveness to salinity. It was observed that monocotyledonous species, especially those belonging to the family Poaceae, are more sensitive to salinity than dicotyledonous ones [157], but within each of these two large groups of plants, a wide range in salt stress responses is displayed. For example, among monocots, rice is the most sensitive and barley the most tolerant [158,159].

Plants have evolved different mechanisms to cope with salt stress. Munns and Tester [6] suggested three main mechanisms: osmotic stress tolerance, ion exclusion, and tissue tolerance. At first, plants respond to salt stress with osmotic adjustments in order to maintain cell turgor, which is necessary to the right functioning of the cell [130,160]. The increased soil salinity causes a decrease in the water potential, inducing root cells to release water into a hypertonic medium. In this context, plants synthesize osmolytes (such as proline, glycine-betaine, and soluble sugars) to mitigate the effects of high salt concentration, counterbalancing osmotic impairment [130,161,162]. Among these osmolytes, proline and glycine-betaine are the most efficient compatible solutes. Due to its ability to be accumulated at high concentrations in response to saline stress, proline can be used as a marker of salt toxicity [163]. Glycine-betaine acts mainly in protecting antioxidant enzymes and PSII [164]. In addition to their osmotic function, osmolytes have been reported to have also a ROS scavenging function [165,166]. During stress exposure, plants try to maintain also the turgor of mesophyll cells in leaves, avoiding the translocation of toxic ions from root to shoot and regulating leaf transpiration. Abscisic acid (ABA) plays a key role in reducing the transpiration rate within a few hours. Its production occurs in response to high salinity at the root level, then it is transported via xylem to leaves, also inducing stomatal closure [3,167]. A lower water loss has been found in species with a certain tolerance to salt. In fact, halophytes generally show a reduced transpiration, if compared to glycophytes [168].

The maintenance of the optimal cytosolic K^+^/Na^+^ ratio is of primary importance to protect cellular functions [169]. As reported before, an increasing uptake of Na^+^ due to salt stress induces an imbalance of K^+^ transport in root cells. Na^+^ ions in excess need to be extruded or compartmentalized in the vacuole not to become toxic [130,170]. Ion sequestration in the vacuole, or exclusion from the cytosol, are two tolerance mechanisms adopted by plants subjected to salinity stress. In order to allow the “normal” metabolic and enzymatic function, plant cells continue to store toxic ions in the vacuole until saturation level. This compartmentalization process effectively reduces the cytoplasmic concentration of Na^+^ [171]. On the other side, the plasma membrane Na^+^/H^+^ antiporters translocate the excess of Na^+^ outside the cell, into the extracellular region, in order to maintain a low concentration of Na^+^ in the cell [172]. The salt overly sensitive (SOS) pathway was discovered in *A. thaliana* and includes *SOS1*, *SOS2*, *SOS3* genes. The SOS pathway has a key role in maintenance of homeostasis, in cytoskeleton reorganization, and in modification of root architecture [173]. The main gene involved in the transport of Na^+^ is *SOS1*, encoding for a Na^+^/H^+^ antiporter.

## 5. Influence of Salinity and Aridity on Microbial Communities and on Plant Microbial Interactions

Salinity negatively affects the physico-chemical properties of soil such as pH, nutrient availability for plants, cation exchange, and soil organic carbon input, strongly influencing the soil microbial communities and their activity [174,175]. The maintenance of soil organic carbon input is essential for soil biological and ecological functions. Soil salinization is a degradation mechanism that involves the accumulation of water-soluble salts in the upper part of the soil and affects the agricultural yield, the productivity of ecosystems, and therefore economic well-being. Strategies for maintaining agricultural productivity and alleviating water and salt stress include the development of salt tolerant cultivars, the implementation of alternative cultivation systems and specific management practices, such as drip irrigation or soil drainage. Stress can also be reduced by modifying soils with organic substances such as manure, compounds, or straw, which favor the promotion of the growth of microbial biomass. In fact, plants can benefit from the activity of microorganism communities that colonize their root systems: as previously explained, the bacterial production of EPS can help plants to resist drought and salinity, as well as enzymatic activities facilitate the formation of soil aggregates, the stabilization of organic carbon, and the production of hormones and volatile compounds that trigger defense mechanisms and promote growth [174]. The study of the microbiota associated with plants, its characterization and understanding of the role of both bacteria and fungi within the rhizosphere, is one of the main topics in current research.

Microbial communities are influenced by various abiotic and biotic factors. Greenhouse and field studies have shown that soil type, plant genotype, plant developmental stage, and cultivation practices determine the composition and structure of the microbiota [175,176,177,178,179]. Among these factors, the type of soil and, in particular, its physical-chemical properties, are the strongest determinants in the variation of the microbial community: pH is the most critical parameter. Soils with almost neutral pH generally have a greater bacterial diversity than more acidic or more basic soils, such as deserts.

Although the relationship between soil characteristics and its microbial communities present has been extensively studied, the effect of one on the other is largely unknown [180]. Furthermore, the microbiota is not static or passive: plants can actively modulate the construction of their microbiota, especially thanks to the deposition of extraradical exudates [181].

Autochthonous plants of the desert or semi-arid environments are constantly subjected to water and saline stress: these factors limit the number of species that manage to survive and shape the composition of the microbial community [182]. This idea is also confirmed by Fierer [183], based on the analysis of the microbiota in cold deserts, hot deserts, forests, prairies, and tundra. Fierer demonstrated that antibiotic resistance genes were three times less abundant in desert soils than other soils, suggesting that abiotic conditions are more important than competitive interactions in shaping desert microbial communities. Studies on various desert environments reveal both the antifungal [184] and the antibiotic properties [185] for the communities of Actinobacteria.

Investigations regarding bacterial diversity in the semi-arid zone of central Mexico have revealed the predominance of Actinobacteria, Proteobacteria, and Acidobacteria in the rhizosphere of cactus plants [186]. An analysis of the microbial communities in the Atacama desert (Chile) has shown a high number of Actinobacteria, Chloroflexi, low levels of Acidobacteria, and alpha and beta-Proteobacteria [187]. Acidobacteria are typically found in environments that vary in temperature, soil type, and pH. Basic soils such as desert generally have higher relative abundance of Actinobacteria than Acidobacteria [188]. This observation is confirmed by the study on the vineyards of Berlanas [189]: the vineyards have a more acidic soil than the desert, and the Acidobacteria (13.7% and 16.4%) immediately follow the Proteobacteria and the Actinobacteria (almost 50% of the total).

Both beta and alpha-proteobacteria are commonly associated with soils that receive high organic carbon input rates, a condition absent in the desert. Fierer [183] showed that all communities related to the different biomes (cold deserts, warm deserts, forests, grasslands, and tundra) were dominated by Acidobacteria, Actinobacteria, Bacteroidetes, Proteobacteria, Verrucomicrobia, and Gemmatimonadetes, bacterial phyla known to be relatively abundant and omnipresent in the soil. Actinobacteria and Bacteroidetes are generally more abundant in desert soils than in forests, grasslands, and tundra, while Verrucomicrobia and Acidobacteria have shown the opposite trend. Chloroflexi phyla, Cyanobacteria, Firmicutes, and Gemmatimonadetes have also been found in almost all soils, but their relative abundances are highly variable and represent less than 5% of the identified sequences. Firmicutes members, particularly *Bacillus*, are responsible for the production of a wide range of antimicrobial metabolites, enzymes, and surfactants that promote plant growth and induce systemic resistance in plants [190]. It is also possible that the low diversity of desert soils is not directly related to their very high pH levels, but rather to the high salinity. The desert soils are very dry, poor in nutrients, generally have a basic pH, higher than other biomes and the scarcity (or complete absence) of plant biomass reduces the inputs of organic carbon useful for bacterial metabolism. When the amount of organic matter is greater in soil, the stability and diversity of the microbiota is higher too [26]. For example, Gao and coworkers [188], in a study concerning the effect of aridity and dune type (in a desert of Northern China), showed that Firmicutes, Actinobacteria, Proteobacteria, and Acidobacteria were the dominant phyla in all samples of the rhizosphere of *Caragana microphylla*. The increased abundance of Actinobacteria in the rhizosphere soil was mainly caused by the decreased soil pH due to rhizodeposition [188]. The authors stated that the structure of the rhizospheric bacterial community was modulated mainly by soil total organic C, total N, Na^+^, and total P while total organic carbon, electronic conductivity, pH, and total phosphorus were the dominant factors to affect the microbial communities associated to the different dunes. Pereira [191] confirms that the low density of bacteria in the desert, compared to a soil not subjected to water stress, can be due to the previous reported physical-chemical characteristics of the arid soil.

Although the interest in the study of microbial communities occurring in arid environments is increasing, the number of works present in the literature and the results obtained do not yet allow to generalize a common trend. Furthermore, finding a common thread could become even more complicated taking into consideration the different species of plants.

## 6. Application of PGPB in Salt Stressed Soil

There is an increasing request for food worldwide: there are 7.4 billion people in the world, and this value is expected to reach 9.7 billion by 2050 [192]. As a consequence, the exploitation of chemical fertilizers in agriculture is considered as necessary regardless of its side effects on the environment and especially on soil. Even more, as previously explained, environmental abiotic stresses such as drought, high salinity, and high and low temperature have negative impact on agriculture productivity. As an example, it has been recently reported that soil salinization leads to a loss of US$ 27.3 billion [193]; in the same way, drought caused a 5% reduction of the yield in the primary food-producing nations and an annual loss of 17% in harvest yield in the tropics, arid, and semi-arid regions. In this context, in order to protect the sustainability of both the natural and agricultural ecosystem and, at the same time, obtain crop yields that meet the increased food demand, there is urgent need of new and eco-friendly approaches in agricultural practices.

PGPB offer a realistic opportunity in protecting crop yield also in stressed soils, due to their beneficial effect on soil fertility, using different strategy (as previously reported in the above paragraphs). In fact, in the event of environmental stress, rhizobacteria modulate their activities by interfering with the stress tolerance mechanisms in plants. The role of bacteria in the rhizosphere in a context of stress is really difficult to be fully understood. As already mentioned, each plant builds its own microbiota which will be different in relation to the type of soil, the environmental components, and the genetics of plant. Therefore, it is very complex to define how the plant responds to stress on the bases of interaction of the associated PGPB. Here are presented some positive rhizobacteria actions that help plants to adapt to stressful conditions.

Both fungi and bacteria can produce ACC-deaminase and a series of phytohormones to influence the plant endogenous hormone levels and eventually reprogram the plant hormonal signaling. Plants subjected to salinity, drought, and pathogenic stress are known to produce an excess of ethylene, which seriously slows down root development [194]. One of the features of PGPB is that they show the ability to produce ACC deaminase, which catalyzes the conversion of ACC (the precursor of ethylene biosynthesis) to ammonia and α-ketobutyrate. Therefore, plants with reduced ethylene levels would eventually overcome salt-induced growth inhibition by associating with the bacteria that produce ACC deaminase [108]. One of the subsequent effects of this particular type of plant-bacterial association would be the supply of a source of N (ammonia) to plants [195]. Little is known about how these PGPB influence this process, although there is some evidence that PGPB improve plant salt tolerance by altering the state of the endogenous hormone [196]. Studies carried out on pepper in the presence of water stress show that plants treated with ACC rhizobacteria recorded higher efficiency of photosynthetic processes and even higher tissue turgidity. These beneficial effects lead to an increase in biomass and root length of up to 50% compared to uninoculated plants. The most pronounced protection against drought has been obtained with strains of the genera *Achromobacter*, *Klebsiella*, and *Citrobacter*. Considering the ability to colonize the roots of these genera, it is conceivable that this protection activity can also be carried out in field conditions and not only in vitro [197].

PGPB can offer a realistic opportunity in increasing crop yields also in stressed soils due to their beneficial effect on soil fertility, improvement of nutrient uptake by plants, the expression of plant beneficial physiological traits, and reducing soil borne plant diseases [198].

In this paragraph, several examples of applications of PGPB in crop growing in salt or drought stressed soils are provided, referring to the most recent scientific papers.

In their work, Amna et al. [199] assessed that effects of *Bacillus siamensis* PM13, *B. methylotrophicus* PM19, and *Bacillus* sp. PM15, three halotolerant strains, able to synthesize ACC deaminase and EPS, on wheat seed germination and seedling growth under three level of salt stress (4, 8, and 16 dS m^−1^). Although a positive impact of bacteria was observed on both shoot and root development, this effect slowed down as the salinity level increased. The induced salt tolerance detected at moderate salinity stress may be based on EPS or ACC deaminase synthesis but also other physiological traits as P solubilization and IAA production could moderate the extent of the damages induced by NaCl [199].

The *Burkholderia* sp. strain P50 is able to produce ACC deaminase, IAA, EPS, and the osmolyte proline also under salinity stress. Rice seedlings inoculated with the strain P50 and grown in presence of 185 mM NaCl were improved in several morphological and biochemical characteristics, as well as in producing antioxidants able to scavenge ROS. Moreover, the amount of stress ethylene in seedlings exposed to salt stress and treated with *Burkholderia* sp. P50 was reduced compared to non-inoculated plants. The mutant of the strain P50 lacking the ACC deaminase genes was unable to reduce stress ethylene, indicating that the action of the *Burkholderia* sp. P50 strain was related to a decrease of ethylene level [200].

In their work, Chandra et al. [201] assessed the ability to alleviate drought stress in wheat by using strains of *Variovorax paradoxus* RAA3, *Pseudomonas palleroniana* DPB16, and *Pseudomonas* sp. UW4 in a field experiment under irrigated and rainfed conditions. The best response to bacterial inoculation was obtained under rainfed conditions, in which wheat was grown under extensive water stress. Moreover, all the three strains induced grain yield increase, but the most performer strain was *V. paradoxus* RAA3: all the plant growth and yield parameters considered (plant height and tillers/plant, spike length, number of grains/spike, total tillers, 1000 grain weight) in both irrigated and rainfed field conditions were increased after inoculation with the strain RAA3. Although the mechanisms at the base of this effect is not currently known, it is true that *V. paradoxus* RAA3 had the highest ACC deaminase activity among the three bacterial strains [201].

Tomato has a great economic impact worldwide, but its sensitivity to water stress can limit its yield [202]. In their recent study, Brilli et al. [203] inoculated tomato seeds with *P. chlororaphis* subsp. *aureofaciens* M71 and exposed plants to a mild water stress condition. The occurrence of this bacterial strain at the root level induced an increased production of osmolytes, such as proline, and enhanced antioxidant molecule levels in leaves, especially those of superoxide dismutase (SOD) and catalase (CAT) enzymes [203]. This kind of effect may boost the plant tolerance to osmotic stress and, at the same time, maintains low ROS levels for signaling purposes. This fact allows the plant to give a quick response to ROS produced by the plant itself during environmental stresses [204]. Therefore, it is suggested that *P. chlororaphis* acted as a ‘priming stimulus’ triggering in inoculated tomatoes enhanced tolerance to water stress [205]. Moreover, an increased content of ABA in leaves was observed in plants inoculated with M71 grown under water stress: this is an interesting effect induced by the bacterial strain since high ABA levels result in stomatal closure, that in turn, has a positive feedback on water use efficiency. As mentioned above, many microbial groups secrete phytohormones, such as IAA, ABA, cytokinins (Cyt), and gibberellic acids (GA) [206]. In the study of Bianco and Defez, it is highlighted how the *Sinorhizobium meliloti* (alpha Proteobacteria) strain produces IAA, leading to an improvement in the growth of *Medicago truncatula* in saline soils [207]. Salt tolerance probably increases in response to stimulation of root proliferation as suggested by Dodd and Pérez-Alfocea [202]. Under saline or normal conditions, PGPB inoculation often decreased the accumulation of ABA in roots and has even significantly altered the remote signaling of phloematic ABA shoot-to-root flows and xylematic root-to-shoot flows [208], flows that can mitigate the adaptation of plants to water deficit. Recently, two rhizospheric bacteria, *Rhodococcus* sp. (Actinobacteria) and *Novosphingobium* sp. (alpha Proteobacteria), have been shown to be able to metabolize ABA *in vitro* and have contributed at least in part to reducing ABA concentrations in plants [208]. Even more interesting, ABA homeostasis disruption influences the activity of the PGPB.

Furthermore, from Porcel’s study, it emerges how *Bacillus megaterium* can promote tomato growth by inhibiting mutant plants deficient in ABA [209]. In conclusion, these results suggest that the PGPB that produce and/or metabolize ABA in general will act differently in modulating the ABA state of plants and therefore could cause variable responses of plants to salinity stress.

PGPB can improve the absorption of nutrients in plants. *Achromobacter piechaudii*, when inoculated in *Solanum lycopersicum*, exposed to high salinity conditions, increases the acquisition of P and K and improves efficiency in the use of water [210]. Inoculation with *Bacillus aquimaris* significantly increase N, P, and K concentration in leaves of wheat, cultivated in field under high saline stress [211]. These results highlight PGPB ability to solubilize insoluble P and to fix atmospheric N in a non-symbiotic way. Similarly, PGPB are able to release siderophores in order to eliminate Fe, then ferri-siderophore can be easily accessible to plants [212].

A useful mechanism for maintaining ionic homeostasis in plants when subjected to saline conditions is expressed by the synthesis of EPS which binds toxic Na and limits its inflow into the roots. EPS can also promote biofilm formation on root surfaces, thereby limiting the flow of Na^+^. Recently, EPS production has been identified as involved in the beneficial effect of *B. amyloliquefaciens* FZB42 in protecting *A. thaliana* from drought stress [213]. The wild-type strain FZB42 promotes plant growth and drought tolerance by enhancing the plant survival rate, the plant biomass, root length, and branching. The EPS deficient mutant of this bacterial strain, once inoculated on the plant root, boosted cellular defense responses such as the level of proline and the activities of SOD and peroxidase and modulated the synthesis of ethylene and jasmonate.

Similar results were obtained in sunflower inoculated with the EPS producer *Pseudomonas putida* GAP-P45 [214], cowpea colonized by *Azospirillum* [215], and foxtail millet treated with *P. fluorescens* DR7 [216].

In case of prolonged exposure to high concentrations, Na^+^ moves from the roots to the breathable leaves where it can increase toxicity. At this point, volatile organic compounds (VOCs) produced by PGPB can be useful, inducing HKT1 gene expression in the shoots, which codes for a high affinity transporter protein of K, expressed in xylem parenchyma cells, that allow to maintain Na^+^ homeostasis. VOCs also induce the reduction of HKT1 in the roots, limiting the entry of Na^+^ and facilitating the recirculation of Na^+^ from bud to root. HKT1 then mediates the loading of Na^+^ into the phloem lymph in the shoots and the discharge into the roots, thus removing large quantities of this ion from the shoots [206].

PGPB positively regulate the expression of genes that code for the integral proteins of the plasma membrane (PIPs) and the activity of aquaporins, for an efficient absorption of water in stressed plants. Analysis of the gene expression of *Pantoea agglomerans* and *Zea mays* roots infected with *B. megaterium* showed that the PIP2 and ZmPIP1-1 genes were up-regulated under saline stress conditions, helping to increase the values of hydraulic conductance in inoculated plants [217]. In a parallel study, inoculation with *Azospirillum brasilense* triggered the transcription of HvPIP2-1 in the seedlings of *Hordeum vulgare* [218]. Furthermore, proteomic analysis revealed that the expression levels of different proteins involved in photosynthesis, antioxidant processes, membrane transport, and pathogenesis-related responses were altered in the presence of PGPB [219].

Almost all PGPB are able to boost antioxidant systems contributing to the scavenging of ROS produced in case of stress, which can alter fatty acids, amino acids, pigments, and other biomolecules of plant cells. The expression of ROS elimination genes, such as SOD, CAT, ascorbate peroxidase (APX), dehydroascorbate reductase (DHAR), and glutathione reductase (GR), increases in PGPB-treated *Solanum tuberosum*, under conditions of abiotic stress [220]. Even after short-term treatment with *Enterobacter* sp., the expression of saline stress sensitive genes related to proline biosynthesis has been up-regulated in *Arabidopsis* [221]. The production of some important osmolites, such as proline and polyamines, is promoted not only in plants subjected to saline and water stress, but also in those subject to microbial infection.

In the case of saline stress, Yuan’s work highlighted that there is an increase in the production of salicylic and jasmonic acid in the soil [222], which seems to be effective in producing tolerance. Many PGPB produce VOCs that interfere with normal hormonal production in the plant, allowing the activation of the defense mechanisms dependent on these hormones. The way in which VOCs work is partially unknown and the study is made difficult since they do not act as individual compounds, but often act synergistically. Endophytes also produce some effectors, including plant hormones, signaling molecules, and microRNAs, for the activation of signaling pathways and often significantly promote metabolic efficiency, altering the ratio between upregulated/downregulated genes. Other classes of endophytes produce some bioactive compounds (melanin, mannitol, and trehalose) to relieve the abiotic stress of plants [206].

In 2016, Tiwari [223] examined the role of *Pseudomonas putida* MTCC5279 inoculated in *Cicer arietinum* plants and showed positive actions against drought stress, including modulation and maintenance of membrane integrity, the accumulation of osmolites (proline, glycine betaine), the ability to wash ROS with transcription of the SOD, CAT genes, APX, and glutathione-S-transferase (GST), the differential expression of genes involved in the biosynthesis of ethylene, salicylic acid, the activation of jasmonic acid, and the greater production of late embryogenesis abundant (LEA) proteins and dehydrins (DHN).

The application of thuricin 17 produced by *Bacillus thuringiensis* NEB17 to soybeans (*Glycine max*) in conditions of water deficit has led to the modification of the root structures, an increase in the biomass of the roots and nodules, in the length of the roots, an increase in ABA, and the total N content in the roots [224].

Wang et al. [225] describe how pea plants inoculated with *Variovorax paradoxus* 5C-2, which produce ACC deaminases, resist saline stress. *V. paradoxus* involves an increase in photosynthetic rate, electron transport, balanced ionic homeostasis through an increase in K^+^ flow to the shoots and deposition of Na^+^ on the roots, a decrease in stomatal resistance and an equilibrium pressure of the xylem. In addition, there is also an increase in biomass at 70 and 130 mM NaCl [225].

Finally, wheat plants (*Triticum aestivum*) inoculated with *Dietzia natronolimnaea* showed an upregulation of the genes involved in the ABA signaling cascade, in the SOS pathway, in ion transporters and in antioxidant enzymes; stress tolerance is induced by the modulation of a complex network of gene families [226].

## 7. Conclusions

It has been estimated that the world’s population reached 7.4 billion and is promptly going to exceed 9 billion [192]. The survival of this huge amount of people is dependent on agricultural products. While the food demand is increasing, the soil quality is reducing due to the intensive use of chemicals and to other different stresses such as salinity and drought. Even more, there is a diffuse reluctance by consumers to embrace the use of genetically modified plants as foods. In this scenario, the exploitation of beneficial microorganisms able to improve plant tolerance and growth in stressed environments may represent an alternative whose aim is to reduce the worldwide dependence on chemicals in agricultural practices and at the same time, enhance the yield potential. Sustainable agriculture should take advantage of the potential of PGPB strains that can be used as biofertilizer, biostimulant, and biocontrol agents. However, we are not completely ready as we still need to make an effort in order to disentangle several variables as the best commercial formulation, the survival of the bacterial strains in field conditions, the specificity against the host plant. For these reasons, studies regarding the impact of salinity or drought on soil bacterial communities are essential in order to understand the mechanisms at the base of the bacterial and plant survival in these kinds of environments, as well as the possible interactions between the plant and its microbiota.
